# Two Cases of Idiopathic Middle Meningeal Arteriovenous Fistula Initially Diagnosed by Magnetic Resonance Angiography

**DOI:** 10.7759/cureus.35501

**Published:** 2023-02-26

**Authors:** Hiroshi Kondo, Yoshihiro Kiura, Sayuru Tsuyuguchi, Hideki Satoh, Atsushi Tominaga

**Affiliations:** 1 Neurosurgery and Neuroendovascular Therapy, Hiroshima Prefectural Hospital, Hiroshima, JPN; 2 Neurosurgery and Endovascular Therapy, Hiroshima Prefectural Hospital, Hiroshima, JPN; 3 Neurosurgery, Satoh Neurosurgical Clinic, Hiroshima, JPN

**Keywords:** mmavf, diagnosis, magnetic resonance angiography, pulsatile tinnitus, middle meningeal arteriovenous fistula, idiopathic

## Abstract

Reports of middle meningeal arteriovenous fistula (MMAVF) are relatively rare, and reports of idiopathic MMAVF are extremely rare. In the past, diagnoses of MMAVF have been confirmed by cerebral angiography, but magnetic resonance angiography (MRA) resolution is improving. Here, we report two cases of idiopathic MMAVF that were diagnosed by unreconstructed time-of-flight MRA (MRA-TOF) and successfully treated by trans-arterial embolisation with endovascular treatment. Both patients suffered from pulsatile tinnitus, and MRI was performed. Two dilated vessels were revealed in the middle temporal fossa by unreconstructed MRA-TOF imaging. These dilated vessels were thought to be the middle meningeal artery and middle meningeal vein; therefore, we diagnosed both patients with MMAVF. Following angiography, both patients had coil embolisation with endovascular treatment, and their conditions improved. In cases of idiopathic MMAVF without a history of trauma, brain surgery, or endovascular surgery, unreconstructed MRA-TOF may be useful as a primary diagnostic tool, and endovascular treatment before bleeding may produce better outcomes.

## Introduction

Most middle meningeal arteriovenous fistula (MMAVF) cases are post-traumatic MMAVF [1-6] and sometimes are iatrogenic MMAVF [7-11]. Reports of idiopathic MMAVF are extremely rare [4,12]. On the other hand, MMAVF diagnoses are typically confirmed by cerebral angiography, a relatively invasive examination. However, diagnostic imaging technology has been progressing rapidly, allowing clinicians to diagnose lesions that have been historically difficult to visualise. Herein, we report two cases of idiopathic MMAVF with no history of trauma, brain surgery, or endovascular surgery, diagnosed by unreconstructed time-of-flight magnetic resonance angiography (MRA-TOF), a minimally invasive test. Both patients were successfully treated with trans-arterial coil embolisation from the draining vein via the shunting point to the feeding artery.

## Case presentation

Case 1

**Figure 1:**
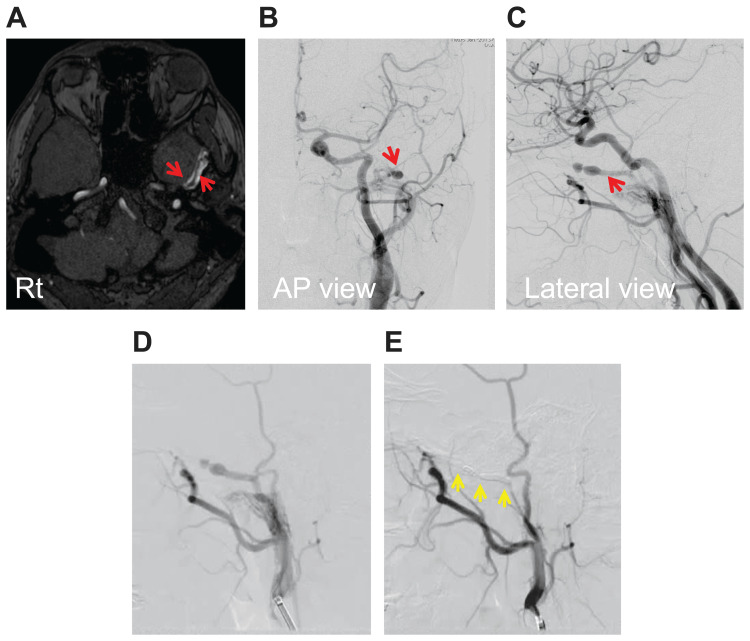
Images of case 1 (A) MRA: Unreconstructed MRA-TOF showed two dilated vessels thought to be MMA and MMV at the middle fossa (two red arrows). (B, C) Preoperative cerebral angiography: Angiography revealed the existence of MMAVF with varices (red arrows) in the anteroposterior (B) and lateral view (C). (D, E) Intraprocedural cerebral angiography: The findings during endovascular treatment, before (D) and after coil embolisation (E). After the procedure, angiography showed complete occlusion with a coil (yellow arrows) and MMAVF disappeared (E). MRA-TOF: time-of-flight imaging of magnetic resonance angiography; MMA: middle meningeal artery; MMV: middle meningeal vein; MMAVF: middle meningeal arteriovenous fistula; AP: anteroposterior.

A 31-year-old male with no history of head trauma, head surgery, or endovascular treatment and with no risk factors for the development of fistula (obesity, hypertension, smoking, and administration of antithrombotic drugs) suffered from pulsatile tinnitus and went to a nearby clinic where a magnetic resonance imaging (MRI) was performed. Two dilated vessels, namely, the middle meningeal artery (MMA) and middle meningeal vein (MMV), parallel to the MMA were revealed in the middle temporal fossa by unreconstructed MRA-TOF (Figure 1A). The patient was transferred to our hospital. Cerebral angiography revealed left MMAVF and varices (Figures 1B, 1C), so trans-arterial coil embolisation with endovascular treatment was performed. A 4 French (Fr) guiding sheath (Fubuki, ASAHI INTECC CO, Aichi, Japan) was placed in the external carotid artery (ECA) using an inner catheter (JB2, Medikit Co Ltd., Tokyo, Japan) and a 0.035-inch guide wire (Radifocus Guidewire, Terumo Interventional Systems, Tokyo, Japan) as a coaxial system, via the right femoral artery. An angiography was performed and MMAVF was confirmed. After an occlusion balloon (Scepter XC, 4 × 11 mm; Terumo Interventional Systems, Tokyo, Japan) was placed in the MMA and inflated, angiography was performed and MMAVF was not shown and thought to have a single feeder. Then, a microcatheter (Excelsior XT 17, Stryker, Kalamazoo, MI, USA) was guided to the MMV, which was the draining vein of MMAVF, and coil embolisation was performed from the MMV to the MMA through a shunt point (Figures 1D, 1E). MMAVF was completely treated by endovascular treatment with coils and its disappearance was confirmed on angiography, and the patient’s symptoms quickly disappeared. No recurrence was observed in the two-year follow-up.

Case 2

**Figure 2:**
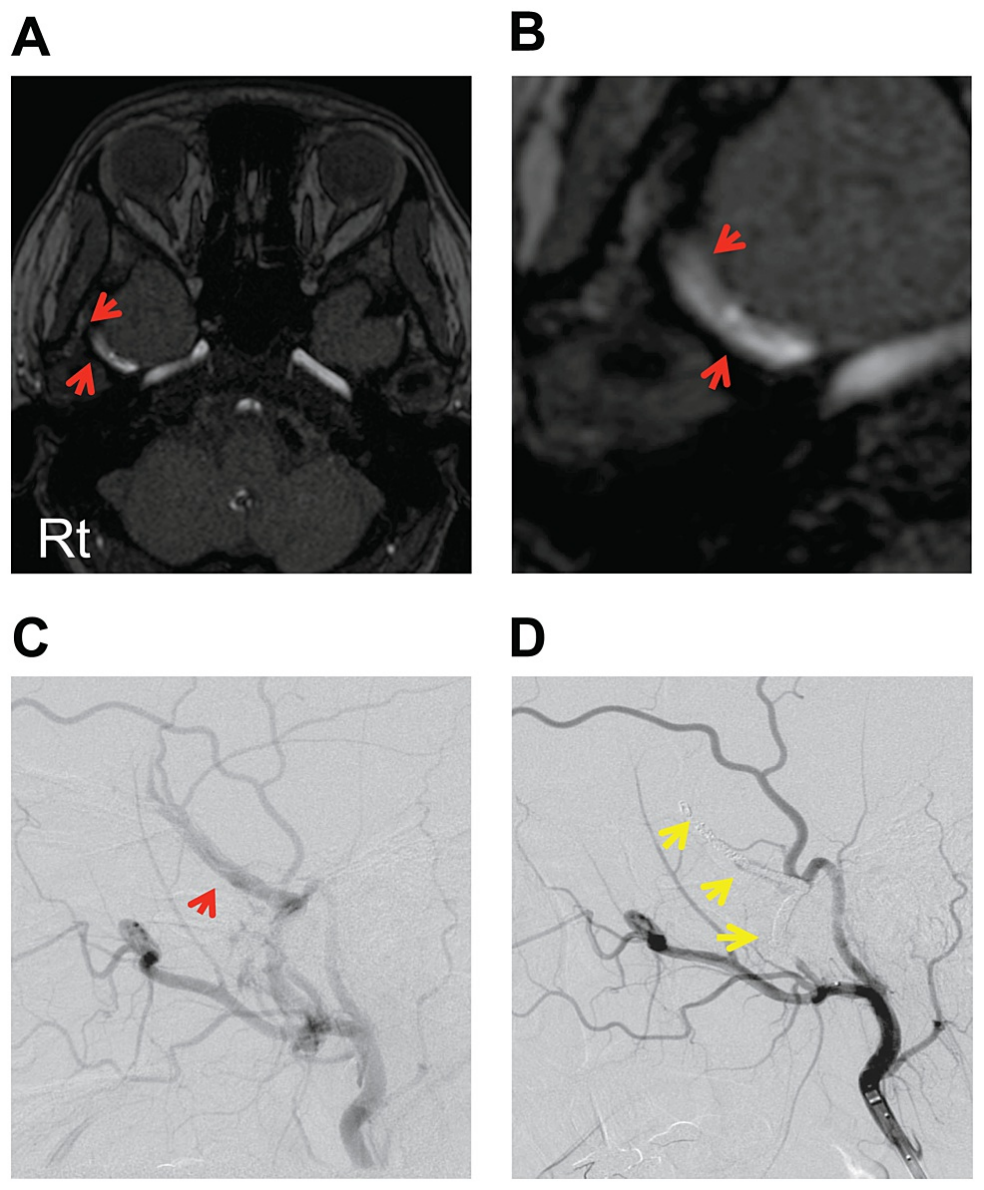
Images of case 2 (A, B) MRA: Two dilated vessels (red arrows) thought to be MMA and MMV were revealed by unreconstructed MRA-TOF in the temporal fossa. (C, D) Intraprocedural angiography: Angiography findings during endovascular treatment, before (C) and after coil embolisation (D). After treatment, MMAVF was completely occluded with a coil (yellow arrows) (D). MRA-TOF: time-of-flight imaging of magnetic resonance angiography; MMA: middle meningeal artery; MMV: middle meningeal vein; MMAVF: middle meningeal arteriovenous fistula; AP: anteroposterior.

A 28-year-old female without a history of head trauma, head surgery, or endovascular treatment and with no risk factors for the development of fistula (obesity, hypertension, smoking, and administration of antithrombotic drugs) had an MRI for pulsatile tinnitus and headache at a nearby neurosurgical clinic. Unreconstructed MRI-TOF showed two dilated vessels, thought to be MMA and MMV, in the middle temporal fossa (Figures 2A, 2B). MMAVF was the primary diagnosis. Angiography revealed MMAVF (Figure 2C), and coil embolisation with endovascular treatment was performed. As with case 1, a 4 Fr guiding sheath was placed in the left ECA using the coaxial system with a 4 Fr inner catheter and 0.035-inch guide wire. An occlusion balloon was guided in the MMA and inflated, and angiography revealed MMAVF with a single feeder. Then, a microcatheter was guided in the MMV through the fistula with a micro guide wire and MMAVF was occluded by coils from MMV, which was the draining vein of MMAVF, to MMA through the shunt point. Complete occlusion of MMAVF was shown by angiography (Figures 2C, 2D), and the patient’s symptoms disappeared soon after the procedure. No recurrence was observed in the one-year follow-up.

## Discussion

Most MMAVF case reports are traumatic [1-6] or iatrogenic [7-11] MMAVF. Anatomically, the MMA and MMV run parallel to the surface of the dura matter [5], and as a histological feature, the media is deficient at the intracranial part [13]. Therefore, it has been reported that the vessel wall injury of MMA caused by head trauma, craniotomy [10], or interventional surgery [7,8] can cause MMAVF. However, there are a few reports of idiopathic MMAVF with no history of head trauma, craniotomy, or interventional surgery [4,12]. In these reports, all cases presented with intracranial haemorrhage. In the present report, two patients without intracerebral haemorrhage suffered from pulsatile tinnitus caused by idiopathic MMAVF. Idiopathic MMAVF may be an alternative diagnosis for pulsatile tinnitus. MMAVF is typically diagnosed by angiography, which is performed to find the cause of the intracranial haemorrhage. However, in recent years, diagnostic imaging technology has made remarkable progress and can detect lesions that were previously difficult to visualise by MRI. Tokairin et al. [14] reported that MRA-TOF and magnetic resonance angiography spin labelling were useful to diagnose MMAVF caused by contrecoup injury. In the present cases, dilated MMA and MMV were detected by the minimally invasive unreconstructed MRA-TOF. Therefore, unreconstructed MRA-TOF may be useful to diagnose MMAVF, especially when the patient presents with a minor symptom such as pulsatile tinnitus. There have been no summarised reports on the natural course of MMAVF, so no standard strategy has been proposed for its therapeutic indication. There are rare reports of MMAVF spontaneously disappearing [11,12]. On the other hand, Rami et al. [4] reported nine cases of MMAVF, which were all caused by intracranial haemorrhage, and mentioned that both traumatic and idiopathic MMAVF should be considered high-risk lesions that require treatment. Additionally, Kitahara et al. [6] reported a case of cavernous sinus syndrome that occurred by the drainage root of MMAVF. In the two present cases, although the only symptom was pulsatile tinnitus, there was a possibility of intracranial bleeding. It is thought that MMAVF was treated successfully through early treatment. In recent reports [2-4,6-8], good obstructions of MMAVF were achieved by embolisation with endovascular treatment, although the embolic materials were different, such as polyvinyl alcohol [7], coil [4,6], n-butyl-2-cyanoacrylate (NBCA) [4], coil + NBCA [8], Onyx [4], and coil + Onyx [2]. In this report, positive results were obtained by coil embolisation with endovascular treatment from MMV to MMA through a shunt point. However, MMAVF, especially idiopathic MMAVF, is a rare disease, and it is necessary to examine its course, diagnosis method, treatment indication, and treatment procedure in more cases.

## Conclusions

MMAVF, specifically idiopathic MMAVF, is a relatively rare lesion with no history of head trauma, head surgery, or endovascular treatment. Because MMAVF may be a high-risk lesion that causes intracranial bleeding, a quick diagnosis is important. It was thought that reviewing unreconstructed MRA-TOF imaging from which the ECA has not been removed may be valuable for a thorough evaluation. Embolisation with endovascular treatment is thought to be useful for MMAVF.
